# Auditory and motor imagery modulate learning in music performance

**DOI:** 10.3389/fnhum.2013.00320

**Published:** 2013-07-01

**Authors:** Rachel M. Brown, Caroline Palmer

**Affiliations:** Department of Psychology, McGill UniversityMontreal, QC, Canada

**Keywords:** sensorimotor learning, auditory imagery, motor imagery, individual differences, music performance

## Abstract

Skilled performers such as athletes or musicians can improve their performance by imagining the actions or sensory outcomes associated with their skill. Performers vary widely in their auditory and motor imagery abilities, and these individual differences influence sensorimotor learning. It is unknown whether imagery abilities influence both memory encoding and retrieval. We examined how auditory and motor imagery abilities influence musicians' encoding (during Learning, as they practiced novel melodies), and retrieval (during Recall of those melodies). Pianists learned melodies by listening without performing (auditory learning) or performing without sound (motor learning); following Learning, pianists performed the melodies from memory with auditory feedback (Recall). During either Learning (Experiment 1) or Recall (Experiment 2), pianists experienced either auditory interference, motor interference, or no interference. Pitch accuracy (percentage of correct pitches produced) and temporal regularity (variability of quarter-note interonset intervals) were measured at Recall. Independent tests measured auditory and motor imagery skills. Pianists' pitch accuracy was higher following auditory learning than following motor learning and lower in motor interference conditions (Experiments 1 and 2). Both auditory and motor imagery skills improved pitch accuracy overall. Auditory imagery skills modulated pitch accuracy encoding (Experiment 1): Higher auditory imagery skill corresponded to higher pitch accuracy following auditory learning with auditory or motor interference, and following motor learning with motor or no interference. These findings suggest that auditory imagery abilities decrease vulnerability to interference and compensate for missing auditory feedback at encoding. Auditory imagery skills also influenced temporal regularity at retrieval (Experiment 2): Higher auditory imagery skill predicted greater temporal regularity during Recall in the presence of auditory interference. Motor imagery aided pitch accuracy overall when interference conditions were manipulated at encoding (Experiment 1) but not at retrieval (Experiment 2). Thus, skilled performers' imagery abilities had distinct influences on encoding and retrieval of musical sequences.

## Introduction

Skilled performance in sensorimotor tasks such as athletic or music performance involves a close coupling of actions with sensory outcomes, both of which must be learned in order to achieve optimal performance. Skilled performers can improve their productions using mental imagery: a subjective experience of the sensory outcomes and/or actions associated with a skill, in the absence of stimulus events or performed actions (Coffman, 1990; Driskell et al., 1994; Roure et al., 1999; Jeannerod, 2001; Zatorre and Halpern, 2005; Hubbard, 2010). Performers engage similar brain regions while imagining the sensory outcomes or actions associated with their skills as while perceiving the sensory outcomes or physically performing the actions, respectively (Jeannerod, 2001; Lotze et al., 2003; Meister et al., 2004; Lotze and Halsband, 2006; Baumann et al., 2007). Furthermore, mental practice and physical practice yield similar changes in neural response (Pascual-Leone et al., 1995; Jackson et al., 2003). Skilled performers such as musicians vary widely in their ability to imagine the sensory outcomes and actions associated with their skill (Brodsky et al., 2003; Highben and Palmer, 2004; Brown and Palmer, 2012). These individual differences in imagery abilities modulate performers' memory for music when auditory or motor information is missing or altered while they learn that music (Highben and Palmer, 2004; Brown and Palmer, 2012). Imagery may therefore enable musicians to generate the experience of sound or motor information that is altered or absent during encoding (Brown and Palmer, 2012). Imagery abilities may also influence how performers retrieve from memory previously-encoded music; this question remains untested. The cognitive processes underlying imagery abilities and how they apply to sensorimotor learning and memory are not yet well understood. We examine how mental imagery abilities aid musicians' encoding and retrieval of music.

Substantial evidence suggests that auditory imagery engages cognitive processes similar to those engaged by auditory perception (for review, see Hubbard, 2010). People make similar judgments about the same sounds when perceived and when imagined, and their judgments of imagined familiar sounds reflect the perceptual characteristics of those sounds. Listeners show similar pitch acuity for heard and imagined tones (Janata and Paroo, 2006), they judge the pitch height of perceived and imagined environmental sounds to be similar (Intons-Peterson et al., 1992), and they rate the qualities of perceived timbres as similar to those of imagined timbres (Halpern et al., 2004). The time it takes listeners to judge the pitch height relation between two tones in a familiar imagined song is proportional to the temporal distance between the two tones in the song (Zatorre and Halpern, 1993). Perceived and imagined sounds also implicate partially-overlapping neural networks, usually involving the secondary auditory cortex (Bunzeck et al., 2005; King, 2006; Voisin, 2006; Daselaar et al., 2010). Auditory imagery for melodies, including imagining the continuation of a familiar melody (Halpern and Zatorre, 1999) or viewing lyrics to a familiar melody and imagining the corresponding melody (Herholz et al., 2012), also engages secondary auditory regions (Zatorre and Halpern, 1993). Thus, auditory imagery may aid sensorimotor learning by recruiting similar cognitive processes to those involved in perceiving auditory outcomes of sensorimotor tasks such as music performance. Performing musicians of Western tonal music usually learn novel musical sequences from a notated score that specifies how the music should sound. Therefore, music-notation-based measures of auditory imagery may be most pertinent to performers' sensorimotor learning. Brodsky et al. (2003) suggested that music notation triggers auditory images in performing musicians who learn novel sequences from notation, and proposed that the silent reading of music notation results in auditory imagery. Highben and Palmer (2004) demonstrated that notation-based tests of auditory imagery, adapted from Wing's (1968) battery of aural skills, discriminated accurate and less accurate learners in conditions without auditory feedback. We use Highben and Palmer's imagery measure in this study to investigate how auditory imagery abilities influence performers' encoding and retrieval of melodies.

Motor imagery also appears to engage cognitive processes similar to those involved in physical (overt) motor behaviors. Substantial evidence demonstrates a close correspondence between the temporal features of executed and imagined movements. People take similar amounts of time to produce and imagine the same actions, such as writing letters or walking a physical distance (Decety and Michel, 1989; Sirigu et al., 1996), and both performed and imagined movements follow similar constraints, such as speed-accuracy trade-offs (Decety and Michel, 1989; Decety and Jeannerod, 1996; Guillot and Collet, 2005). Autonomic responses such as skin resistance and temperature, heart-rate, and respiration also show similar response patterns during movement imagination and execution (Decety et al., 1991; Wuyam et al., 1995; Guillot and Collet, 2005). Motor imagery engages cortical networks involved in movement planning and execution, including the primary motor, premotor, and parietal cortex (for reviews, see Jeannerod, 2001 and Lotze and Halsband, 2006). Imagining movements of different body parts engages regions of the motor cortex that correspond somatotopically to regions engaged by physical movement of those body parts (Stippich et al., 2002). Moreover, motor imagery modulates the excitability of motor cortical-spinal pathways (Fadiga et al., 1999; Stinear et al., 2006). Thus, motor imagery may aid sensorimotor learning by recruiting similar cognitive processes to those involved in performing actions in sensorimotor tasks. Highben and Palmer (2004) developed a motor imagery test, motivated by Gleissner et al. (2000), that measured pianists' ability to detect correspondence between pianists' executed and imagined finger sequences. This measure correlated with pianists' ability to recognize music that was learned in the absence of motor feedback (Brown and Palmer, 2012). We use the same measure in this study to investigate how motor imagery abilities influence performers' encoding and retrieval of melodies.

The use of imagery to engage processes involved in auditory perception and motor production may explain why imagery can be used to improve task performance. Mental practice, or the covert rehearsal of a task without physical practice, can improve performance on sensorimotor tasks, compared to no practice (Driskell et al., 1994). Mental practice such as visualizing or feeling the correct movements in a task has been associated with improvements in athletic performance (Roure et al., 1999), and visualizing and feeling movements, as well as imagining resulting sensory outcomes, has been associated with improvements in music performance (Coffman, 1990). Mental practice also leads to similar neural response changes as physical practice; training on a motor sequence via mental practice can lead to behavioral improvements as well as increased neural activation in orbitofrontal cortex, similar to that shown after physical practice on the same motor sequence (Jackson et al., 2003). Non-musicians trained to play a piano melody showed increased motor cortical excitability in response to cortical stimulation after both physical and mental practice (Pascual-Leone et al., 1995). Such similarities between mental and physical practice outcomes would be expected if imagery and performance share common cognitive resources.

Although mental imagery appears to be common and useful for improving performance on both novel and previously-acquired skills, individuals vary widely in their ability to engage mental imagery. Individual differences in mental imagery have been documented in people's subjective ratings of imagery difficulty, temporal similarity between performed and imagined actions, autonomic response patterns during imagery tasks, and the neural networks engaged during imagery tasks compared to physical tasks (Roure et al., 1999; Munroe et al., 2000; Guillot and Collet, 2005; Guillot et al., 2008). Some individual variation can be explained by expertise. For instance, musicians perform better than non-musicians on musical imagery tasks such as imagining the continuation of melodies or comparing the heights of pitches in imagined familiar songs (Aleman et al., 2000; Herholz et al., 2008). Musicians also perform better than non-musicians on non-musical auditory imagery tasks, such as comparing acoustic characteristics of common sounds (Aleman et al., 2000). Musicians also engage sensorimotor regions (dPMC, SMA) more than non-musicians when imagining either the sensory outcomes or motor movements associated with music performance (Baumann et al., 2007). These findings suggest that imagery may recruit acquired sensorimotor associations formed in skilled performance. Beyond group differences in expertise, variability in imagery abilities has also been demonstrated among individual skilled performers. Musicians differ in their ability to imagine how music sounds based on music notation (Brodsky et al., 2003), even though reading notated music is standard training for Western-trained musicians. Musicians also vary in their ability to imagine a series of motor movements associated with performance (Brown and Palmer, 2012). Thus, even experienced performers demonstrate wide variation in their ability to imagine the sensory outcomes and movements that are relevant to their skill.

What advantages could imagery abilities confer on encoding and retrieval in music performance? First, imagery abilities could compensate for missing auditory or motor information during music encoding by filling in the subjective experience of the missing information. Pianists skilled in auditory imagery recalled and recognized novel music that was learned in the absence of auditory feedback better than pianists less skilled in auditory imagery (Highben and Palmer, 2004; Brown and Palmer, 2012). Similarly, pianists who were skilled in motor imagery recognized music learned in the absence of motor movements (without performing the music) better than pianists who were less skilled in motor imagery (Brown and Palmer, 2012). Second, imagery abilities could influence music encoding by modulating sensitivity to interfering or irrelevant information. Pianists with high auditory imagery skill had worse recognition for music that was previously learned while pianists performed along with computerized recordings of that music than pianists with poorer auditory imagery skill (Brown and Palmer, 2012); this suggests that skilled auditory imagers experienced more interference than less-skilled auditory imagers when performing with sounds that mismatched their own auditory feedback, and this interference disrupted music encoding. Irrelevant tasks have been shown to disrupt imagery vividness (Baddeley and Andrade, 2000), suggesting that imagery abilities may increase sensitivity to interference at encoding. Alternatively, imagery skill may confer an enhanced working memory capacity (Baddeley and Andrade, 2000), as suggested by previous correspondences between imagery ability and working memory measures (Sims and Hegarty, 1997), that increases an ability to inhibit irrelevant information. These alternatives have not yet been directly tested. Third, imagery abilities could influence how performers learn the temporal requirements of performing melodies. Previous studies of imagery abilities have focused primarily on how these abilities influence pitch accuracy of performance. Imagery abilities may also influence how performers learn the temporal features of music. Musicians' auditory imagery abilities (as measured by the ability to imagine single pitches or continuations of pitch or temporal sequences) correlated with temporal synchronization abilities (Pecenka and Keller, 2009), suggesting that imagery abilities may influence performers' sensitivity to temporal regularities. Finally, imagery abilities may influence not only encoding processes but also retrieval processes; this remains an open question, as previous studies examining the influences of imagery on music learning and memory have manipulated conditions during encoding but not during retrieval (Brodsky et al., 2003; Highben and Palmer, 2004; Brown and Palmer, 2012). We test here how auditory imagery and motor imagery abilities help performers encode and retrieve the correct pitch sequence (pitch accuracy) and the temporal features of music (temporal regularity).

The current study examines how imagery abilities influence encoding and retrieval of novel musical sequences: specifically, we examine whether imagery abilities compensate for (fill in) missing information or modulate sensitivity to interfering information at encoding and retrieval. Skilled pianists practiced novel melodies in a Learning phase and subsequently performed them from memory in a Recall phase; pitch accuracy and temporal regularity during performance at Recall were compared with measures of pianists' auditory and motor imagery abilities. Pianists learned melodies by listening (without playing) or by playing on a keyboard without sound (during Learning) and they subsequently performed the melodies from memory with auditory feedback (during Recall). In Experiment 1, learning conditions were combined with three possible interference conditions, auditory interference, motor interference, or no interference, during the Learning phase. This design allowed us to examine the influence of missing or interfering auditory or motor information at encoding on subsequent retrieval, as well as how imagery abilities modulated encoding effects. In Experiment 2, interference conditions were presented during Recall. This design allowed us to examine the influence of interfering information at retrieval, as well as how imagery abilities modulated retrieval effects. Independent measures of auditory and motor imagery, based on previous studies (Highben and Palmer, 2004; Brown and Palmer, 2012) examined individual differences in performers' imagery abilities and how they modulated encoding and retrieval effects in both experiments.

## Experiment 1

Experiment 1 addressed three potential influences on sensorimotor encoding of music: type of learning (auditory versus motor), type of interference (auditory versus motor) presented during Learning, and performers' imagery abilities (auditory and motor). Skilled pianists learned melodies in each of six learning-interference conditions that crossed auditory or motor learning with auditory interference, motor interference, or no interference. Participants learned each melody by either listening alone (auditory learning) or performing without auditory feedback (motor learning). During the Learning phase, participants either heard an additional melody (auditory interference), performed an additional motor sequence (motor interference), or received no interference. After learning each melody, participants immediately performed the melody from memory with auditory feedback (Recall). Both pitch accuracy and temporal regularity were measured during the Recall phase, and participants additionally completed independent tests of auditory and motor imagery ability.

The following predictions were tested: (1) If imagery abilities modulate encoding, then imagery abilities should interact with learning and/or interference conditions at encoding; (2) If mental images are used to compensate for missing information at encoding (during the Learning phase), then high auditory imagers should perform more accurately and regularly from memory than low auditory imagers following motor learning, and high motor imagers should perform more accurately and regularly from memory than low motor imagers following auditory learning; (3) If imagery ability increases sensitivity to interference at encoding, as indicated previously in music performance (Brown and Palmer, 2012), then high auditory imagers may perform less accurately and regularly from memory than low auditory imagers following learning with auditory interference, and high motor imagers may perform less accurately and regularly from memory than low motor imagers following learning with motor interference.

## Materials and methods

### Participants

Twenty-four adult pianists (21 females) with a mean age of 21.17 (*SD* = 3.29) years were recruited from the Montreal music community. Pianists had an average of 12.13 (range = 8–18) years of formal, private piano instruction. Participants reported having no speaking, hearing, or learning disorders. No participants reported having absolute (perfect) pitch. Handedness was assessed by self-report. In order to qualify for the experiment, participants performed a sight-reading task which required them to perform a melody from standard musical notation accurately (with no pitch errors) within two trials. The sight-reading melody was similar in length and rhythmic complexity to the melodies used in the experiment (described below).

### Equipment

Participants performed on a Roland RD 700 electronic keyboard with weighted keys. They listened to their performances or to computer-generated stimuli presented through AKG K271 Studio headphones at a comfortable volume. Stimuli were presented via a Roland Soundcanvas SC-55 tone generator. Auditory feedback from the keyboard was controlled and all keystroke responses were recorded in MIDI format using the Ftap program (Finney, 2001) on a Dell PC.

### Stimulus materials

Musical stimuli consisted of 24 novel short melodies composed for a previous study and previously standardized in terms of memorability (Brown and Palmer, 2012). Melodies were about two measures long, in 4/4 meter, and consisted of a single melodic line, to be performed by the right hand. Each melody consisted of a 12-pitch sequence, and the pitches comprising each melody fell within a one-octave range; melodies contained unique pitch sequences in a range of musical keys and simple rhythmic patterns that consisted mainly of quarter notes and eighth notes. Melodies were divided into six sets of four melodies, which were rotated among the six learning-interference conditions. Each set consisted of two melodies in major keys and two in minor keys presented in alternating order; closely-related (relative or parallel) keys were not included in the same set. The 24 melodies were presented one at a time in standard musical notation in all learning conditions. Numbers underneath each notated pitch specified the sequence of finger movements that pianists were instructed to use, to encourage uniformity of performance across all trials for a given melody, and across participants in all conditions. Finger movement sequences were determined by an experienced pianist, and no participants reported that the prescribed movement sequences were disruptive to their performance. Computer-generated recordings as well as auditory feedback from the keyboard were presented with a piano timbre (Soundcanvas: “Piano 1”). The tempo was moderate and was set to a quarter-note (beat) interonset interval (IOI) of 500 milliseconds (ms). Each presentation and/or performance of every melody was preceded by a set of four metronome beats set to a woodblock timbre (Soundcanvas: “Woodblock”). A silence lasting either three or four beats separated each melody presentation from the next set of metronome beats. Computer-generated melodies were presented in all auditory learning conditions with metronomic timing and uniform intensity.

A single additional auditory interference melody was presented in all auditory interference conditions. The auditory interference melody was a unique, isochronous melody in 4/4 meter that was designed to be synchronized with each quarter note beat in the test melodies. The auditory interference melody was always the same length as the test melody; it was also in a higher register than the test melodies (at least one octave higher) and was transposed on each trial to the same musical key as the test melody. The auditory interference melody was presented via computer with metronomic timing and uniform intensity. An additional motor interference sequence, performed by participants in all motor interference conditions, consisted of a five-finger isochronous pattern performed on the piano with the left hand synchronized with each quarter note beat of the notated melody. The pattern was always 1-2-3-4-5-4-3-2-1, with the thumb as “1”, and was always performed on the same set of piano keys (with the thumb on D3 and all fingers on adjacent white keys) with no auditory feedback.

### Design

This study used a within-subjects design. The independent variables were learning condition (auditory or motor) and interference condition (auditory, motor, or none) and the dependent variables measured during Recall were pitch accuracy (the percentage of pitches performed accurately from memory) and temporal variability (the coefficient of variation of quarter-note interonset intervals, or IOIs). Covariates were the auditory and motor imagery ability scores. Participants learned melodies either by listening only (auditory learning) or by performing the melody without sound (motor learning); these conditions were crossed with three possible interference conditions: no interference, auditory interference, or motor interference. This yielded six learning-interference conditions: (1) *auditory learning—no interference*, (2) *auditory learning—auditory interference*, (3) *auditory learning—motor interference*, (4) *motor learning—no interference*, (5) *motor learning—auditory interference*, and (6) *motor learning—motor interference*. Auditory learning consisted of listening to the notated melody, and motor learning consisted of performing the notated melody without sound. Auditory interference consisted of hearing the auditory interference melody (described above) while simultaneously learning the notated melody. Motor interference consisted of performing the motor interference sequence (described above) while simultaneously learning the notated melody. Each participant learned 24 melodies (four melodies per learning condition). Stimuli were rotated among the six learning-interference conditions such that each stimulus was equally represented in each condition across participants. In each learning-interference condition, each melody was learned over six trials with notation, and was then immediately performed from memory (Recall) over four trials without notation, with auditory feedback. The task was blocked by learning-interference condition. Thus, each block of the task contained six Learning trials and four Recall trials for each of four melodies (for a total of 24 Learning trials and 16 Recall trials per block). For each melody, the six Learning trials were always followed immediately by four Recall trials. The order of these conditions was counterbalanced across subjects in a Latin-squares fashion.

### Procedure

Each participant first completed a consent form and then completed a brief test of their sight-reading ability. Participants then completed a musical background questionnaire and tests of auditory and motor imagery ability. The auditory imagery test was adapted from Wing's (1968) battery of aural skills and required participants to detect differences between notated and sounded melodies, presented simultaneously. Two-thirds of the twelve trials contained a single note difference between notated and sounded melodies (for further details, see Highben and Palmer, 2004). The motor imagery test, adapted from Highben and Palmer (2004) and identical to Brown and Palmer (2012), required participants to detect differences between imagined and performed sequences of finger movements. On each of 12 trials, participants first memorized an eight-item finger movement sequence for the left hand from a sequence of pictures; they then viewed a sequence of eight letters (corresponding to piano keys) and performed that eight-note sequence of finger movements with the left hand on a piano keyboard, without hearing sound from the keyboard. Participants reported whether the memorized movement sequence was the same as or different from the performed movement sequence; two-thirds of the trials contained a single finger movement difference between the memorized and performed movement sequences.

Participants then completed the six learning-interference conditions. Participants sat at an electronic keyboard while wearing headphones. Participants first learned and performed a practice melody from memory at the beginning of each task block. In each learning-interference condition, participants did the following for each of four melodies: participants learned a melody with notation over six trials by listening to it or performing it without sound (six Learning trials) and then immediately performed that melody from memory four times without notation and with normal auditory feedback (four Recall trials). Recall conditions were the same across all learning-interference conditions. In the *auditory learning—no interference* condition, participants heard a computerized recording of a melody six times while seeing the notated melody and holding their hands in fists to prevent them from moving their fingers. In the *auditory learning—auditory interference* condition, participants heard a computerized recording of a melody six times while seeing the notated melody and holding their hands in fists; each time participants heard the melody, they also heard the auditory interference melody. In the *auditory learning—motor interference* condition, participants heard a computerized recording of a melody six times while seeing the notated melody and holding their right hand in a fist to prevent them from moving their fingers; each time the participants heard the melody, they would also perform the motor interference task with the left hand, synchronized with each quarter note of the notated melody, while hearing no sound from the left-hand keystrokes. In the *motor learning—no interference* condition, participants performed a melody six times from notation without hearing the sound from the keyboard; participants only heard the first pitch of the melody while they performed the melody on each trial to provide them with an auditory reference as to how the melody would sound. In the *motor learning—auditory interference* condition, participants performed a melody six times from notation without hearing the sound from the keyboard, except for the first pitch of each melody; each time participants performed the melody, they also heard the auditory interference melody. In the *motor learning—motor interference* condition, participants performed a melody six times from notation without hearing the sound from the keyboard, except for the first pitch of each melody; each time the participants performed the melody, they also performed the motor interference task. Participants were always instructed to learn the notated melody. The entire experiment lasted approximately 90 min and participants received a nominal fee.

## Results

### Pitch accuracy during recall

**Figure 1:**
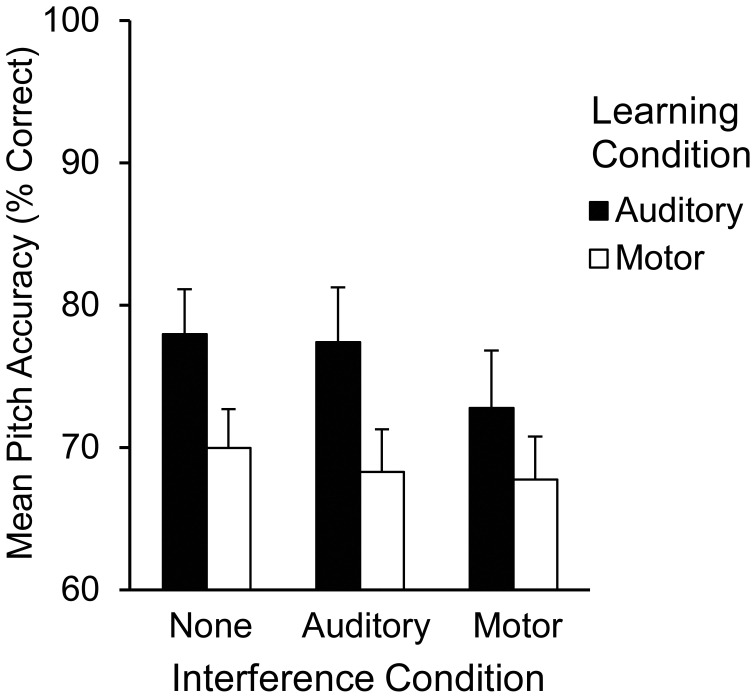
**Mean pitch accuracy (% correct pitches) during Recall by learning condition and interference condition in Experiment 1**.

The alpha level was set at 0.05 for all tests. Pitch accuracy was measured for each Recall trial as the percentage of pitches (out of 12) performed correctly from memory. Mean pitch accuracy is shown in Figure 1 by learning and interference conditions. A two (learning condition: auditory, motor) by three (interference condition: none, auditory, or motor) repeated measures analysis of variance (ANOVA) was performed on pitch accuracy scores. The ANOVA included auditory and motor imagery test scores, each calculated as percent correct out of 12 items, as covariates. A main effect of learning condition [*F*_(1, 20)_ = 17.21, *p* < 0.001] revealed that pitch accuracy following auditory learning (*M* = 76.30%, *SEM* = 2.70) was higher than pitch accuracy following motor learning (*M* = 68.50%, *SEM* = 2.30); there was a marginally significant main effect of interference condition [*F*_(2, 40)_ = 3.02, *p* = 0.06] such that pitch accuracy was higher following no interference (*M* = 74.00%, *SEM* = 2.30) than following motor interference (*M* = 70.20%, *SEM* = 2.60); pitch accuracy following auditory interference (*M* = 72.90%, *SEM* = 2.50) did not differ significantly from pitch accuracy following no or motor interference. Participants' accuracy on the motor interference task was consistently high (96.97% correct key-presses), confirming that they performed the motor interference task correctly. There was no significant interaction between learning and interference conditions. Main effects of auditory imagery test scores [*F*_(1, 20)_ = 10.95, *p* < 0.01] and motor imagery test scores [*F*_(1, 20)_ = 7.91, *p* < 0.05] revealed that higher auditory and motor imagery scores were associated with higher pitch accuracy overall at Recall; there was no significant interaction between auditory and motor imagery. There was a significant three-way interaction between learning condition, interference condition, and the auditory imagery test scores [*F*_(2, 40)_ = 3.47, *p* < 0.05]. This interaction is shown in the parameter estimates (Table 1) for auditory and motor imagery in each condition. As indicated in Table 1, higher auditory imagery scores were associated with higher pitch accuracy following auditory learning with auditory and motor interference and following motor learning with no interference or motor interference. Higher motor imagery scores were associated with higher pitch accuracy following five out of six learning conditions (the sixth condition reached boarderline significance at *p* = 0.07). No other main effects or interactions were found (*p's* > 0.05).

**Table 1:** **Experiment 1: Parameter estimates for auditory and motor imagery effects by condition**.

**Learning**	**Interference**	**Parameter**	**B**	***SE***	***t***	**Sig.**
Auditory	None	Intercept	0.78	0.03	27.47	<0.001
		*Auditory Imagery*	0.21	0.11	2.03	0.056
		**Motor Imagery**	0.41	0.18	2.25	0.036^*^
		Auditory^*^Motor Imagery	0.04	0.73	0.06	0.953
	Auditory	Intercept	0.78	0.03	26.22	<0.001
		**Auditory Imagery**	0.38	0.11	3.46	0.002^*^
		*Motor Imagery*	0.36	0.19	1.91	0.071
		Auditory^*^Motor Imagery	0.65	0.77	0.85	0.406
	Motor	Intercept	0.73	0.03	23.35	<0.001
		**Auditory Imagery**	0.40	0.12	3.47	0.002^*^
		**Motor Imagery**	0.47	0.20	2.34	0.030^*^
		Auditory^*^Motor Imagery	0.41	0.81	0.50	0.621
Motor	None	Intercept	0.70	0.02	31.96	<0.001
		**Auditory Imagery**	0.27	0.08	3.32	0.003^*^
		**Motor Imagery**	0.33	0.14	2.37	0.028^*^
		Auditory^*^Motor Imagery	0.002	0.57	0.003	0.998
	Auditory	Intercept	0.68	0.03	25.34	<0.001
		Auditory Imagery	0.16	0.10	1.60	0.125
		**Motor Imagery**	0.49	0.17	2.82	0.011^*^
		Auditory^*^Motor Imagery	−0.39	0.69	−0.56	0.581
	Motor	Intercept	0.68	0.03	25.34	<0.001
		**Auditory Imagery**	0.25	0.10	2.55	0.019^*^
		**Motor Imagery**	0.41	0.17	2.38	0.027^*^
		Auditory^*^Motor Imagery	−0.45	0.69	−0.66	0.520

**Bold**,

* **p<0.05**;

*Italics, p < 0.10*.

**Figure 2:**
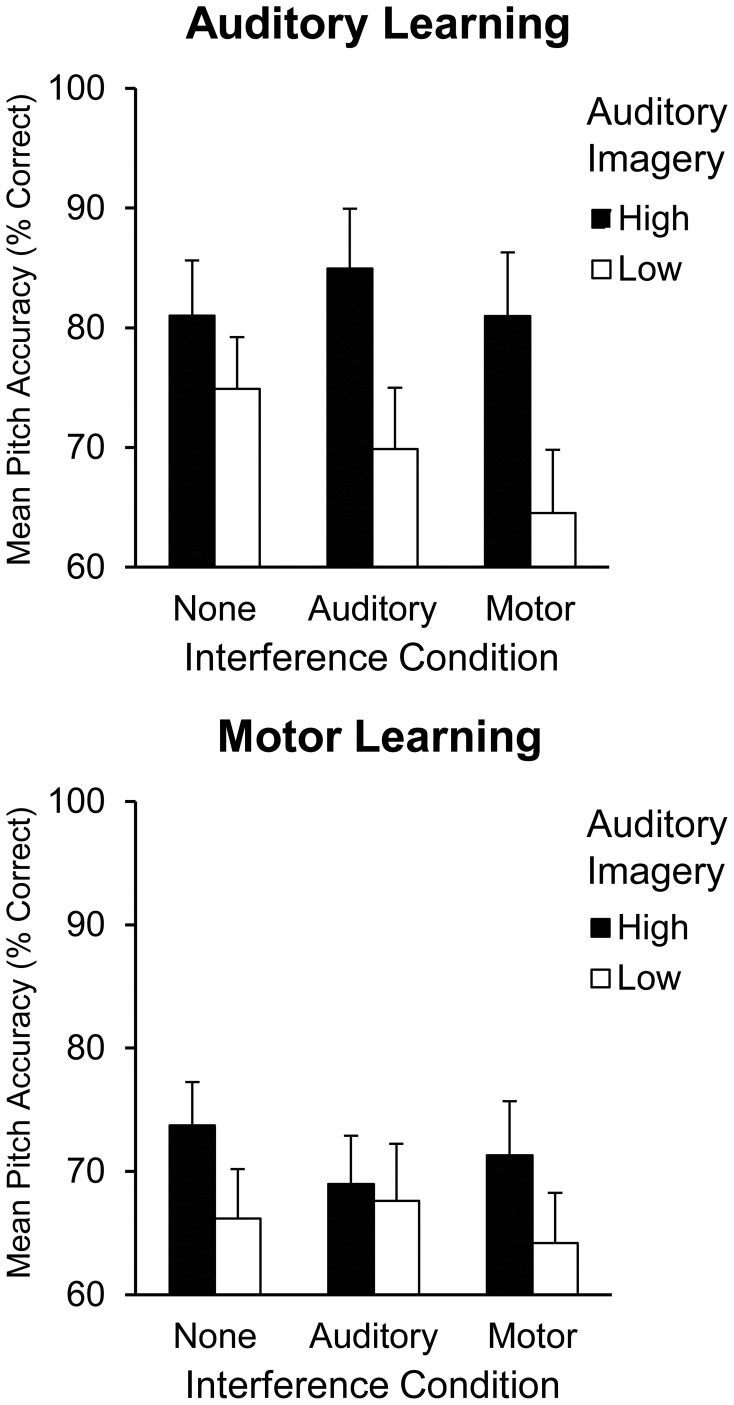
**Mean pitch accuracy (% correct pitches) during Recall by auditory imagery group, learning condition, and interference condition in Experiment 1**.

To further illustrate the interaction between auditory imagery and learning and interference condition, Figure 2 displays high and low auditory imagery groups (divided via a median split, with a median score of 75%) by learning condition (auditory, motor) and interference condition (none, auditory, or motor). A follow-up analysis was conducted to confirm a three-way interaction between auditory imagery group and learning and interference conditions. A two (high and low auditory imagery group) by two (learning condition) by three (interference condition) repeated measures ANOVA confirmed this three-way interaction [*F*_(2, 44)_ = 3.34, *p* < 0.05]; high auditory imagers demonstrated better pitch accuracy than low auditory imagers following auditory learning with auditory interference (high auditory imagers: *M* = 84.94%, *SEM* = 5.01; low auditory imagers: *M* = 69.88%, *SEM* = 5.12) and following auditory learning with motor interference (high auditory imagers: *M* = 80.99%, *SEM* = 5.32; low auditory imagers: *M* = 64.54%, *SEM* = 5.26; *T*ukey's HSD = 10.49, *p* < 0.05).

To further assess the relationship between participants' imagery abilities and other behavioral indices, their auditory and motor imagery scores were correlated with each other, with years of piano instruction, self-rated sight-reading ability, age at which participants started playing piano, and number of hours they currently practice piano; none of the correlations reached significance.

### Temporal variability during recall

Variability of performance timing (coefficient of variation, CV, or standard deviation of quarter-note IOIs, divided by the mean IOI; Schmidt and Lee, 1999) during Recall trials was examined for influences of learning, interference, or imagery abilities. Due to the fact that pitch errors commonly increase timing variability (Palmer and van de Sande, 1993; Pfordresher and Palmer, 2006), a subgroup of 12 subjects with the highest pitch accuracy during Recall trials (mean accuracy per trial = 83.83%) were examined; only pitch-perfect Recall trials were included. This allowed us to examine performance timing separately from the influence of pitch errors in Recall trials. A two (learning condition) by three (interference condition) repeated measures ANOVA on CV measures, with the two imagery test scores as covariates, revealed no significant effects of learning, interference, or imagery scores on CV measures during Recall trials.

## Discussion

Pianists' imagery abilities modulated the influences of learning (auditory or motor) and interference (auditory, motor, or none) on subsequent performance from memory. High auditory and motor imagery skills predicted better pitch accuracy at Recall following all learning-interference conditions. This finding, coupled with a lack of correlation between the auditory and motor imagery scores, suggests that auditory and motor imagery abilities reflect distinct cognitive capacities and contribute differently to performance from memory. Additionally, auditory imagery abilities modulated both learning and interference effects on pitch accuracy at Recall. Highly skilled auditory imagers performed pitches more accurately than less skilled auditory imagers following auditory learning in the presence of either type of interference (auditory or motor). Auditory imagery ability may thus enhance performers' capacity to maintain or manipulate melodies in working memory while performing or hearing additional sequences, thus enhancing auditory encoding in the face of increased task demands. Higher auditory imagery abilities also predicted better pitch accuracy at Recall following motor learning with motor or no interference, suggesting that auditory imagery compensated for missing auditory feedback at learning. Skilled auditory imagers may generate the subjective experience of the missing sound, a process likely subserved by secondary auditory regions that are commonly engaged by auditory imagery (Zatorre and Halpern, 2005). These findings suggest that auditory imagery helps to complete missing information and also protects against interference at encoding. Motor imagery did not interact with learning or interference conditions; instead, higher motor imagery scores predicted better pitch accuracy at Recall across all learning-interference conditions.

Pianists demonstrated better pitch accuracy during Recall following auditory learning than following motor learning. This finding suggests that pianists on average may have relied more on auditory encoding than motor encoding to perform from memory, or that auditory learning followed a faster trajectory than motor learning. Pianists also demonstrated worse pitch accuracy following an additional motor task at encoding. Auditory interference did not disrupt encoding overall; nonetheless, auditory interference interacted similarly with auditory imagery as did motor interference, suggesting that the additional melody may have increased cognitive demands during encoding.

Thus, auditory and motor imagery abilities both had significant effects on pitch accuracy during Recall across all manipulations of learning and interference in Experiment 1. These findings are consistent with previous measures of individual differences in imagery abilities (Highben and Palmer, 2004; Brown and Palmer, 2012) and indicate that auditory imagery abilities predict both general pitch accuracy at Recall and pitch accuracy at Recall following specific learning-interference conditions. Auditory imagery abilities thus appear to modulate music encoding: auditory imagery skill may decrease susceptibility of auditory learning to interference and compensate for missing auditory feedback at encoding. This raises the question of whether auditory and motor imagery abilities also modulate retrieval of auditory-motor sequences. Only encoding conditions were manipulated in Experiment 1 while retrieval conditions were kept constant. Therefore, Experiment 1 was insensitive to effects of imagery abilities on retrieval. Experiment 2 addresses whether auditory or motor imagery abilities modulated retrieval by manipulating interference conditions during Recall and examining how imagery abilities and learning conditions interact with those interference effects.

## Experiment 2

Experiment 2 investigated the influence of type of learning (auditory vs. motor), type of interference at Recall (auditory vs. motor), and performers' imagery abilities (auditory and motor) on performance from memory (Recall). Skilled pianists learned melodies in the same auditory or motor learning conditions; in contrast to Experiment 1, the interference conditions were presented during Recall and not at Learning. This design allowed us to examine the influences of imagery and interference during retrieval; interactions between imagery abilities and interference at Recall would indicate that imagery abilities modulate retrieval. Participants learned each melody by either listening alone (auditory learning) or performing without auditory feedback (motor learning); subsequently, while participants performed the melody from memory with auditory feedback (Recall), they either heard an additional melody (auditory interference), performed an additional motor sequence (motor interference), or did neither (no interference). Both pitch accuracy and temporal regularity were measured at Recall, and participants completed the independent tests of auditory and motor imagery ability. The following predictions were tested: (1) If imagery abilities modulate retrieval, then imagery abilities should interact with interference conditions at Recall; (2) If imagery ability decreases vulnerability to interference during retrieval, then high auditory imagers may demonstrate higher pitch accuracy and temporal regularity at Recall than low auditory imagers in the presence of auditory interference, and high motor imagers may demonstrate higher pitch accuracy and temporal regularity at Recall than low motor imagers in the presence of motor interference. (3) Mental images may still be used to compensate for missing information at encoding; therefore, as found in Experiment 1, high auditory imagers should demonstrate higher pitch accuracy and temporal regularity than low auditory imagers at Recall following motor learning.

## Materials and methods

### Participants

Twenty-four adult pianists (13 females) with a mean age of 22.38 (*SD* = 3.88) years were recruited from the Montreal music community. None of the pianists had participated in Experiment 1. Pianists had an average of 11.34 (range = 8–18) years of formal, private piano instruction. Participants reported having no speaking, hearing, or learning disorders. No participants possessed absolute (perfect) pitch according to self-report. Handedness was assessed by self-report, and there was no difference across experiments in the distribution of left- and right-handed participants. Only participants who accurately performed the same sight-reading test as used in Experiment 1 without any pitch errors within two trials were included in the study.

### Equipment and stimulus materials

All equipment and stimuli used in Experiment 2 were identical to those used in Experiment 1.

### Design

The experimental design and task were identical to those in Experiment 1, with the following exception: the interference conditions were presented during Recall trials, instead of at Learning. Learning and interference conditions were crossed, yielding six learning-interference conditions: (1) *auditory learning—no interference*, (2) *auditory learning—auditory interference*, (3) *auditory learning—motor interference*, (4) *motor learning—no interference*, (5) *motor learning—auditory interference*, and (6) *motor learning—motor interference*.

### Procedure

The procedure was identical to that of Experiment 1. The entire experiment lasted approximately 90 min and participants received a nominal fee.

## Results

### Pitch accuracy during recall

**Figure 3:**
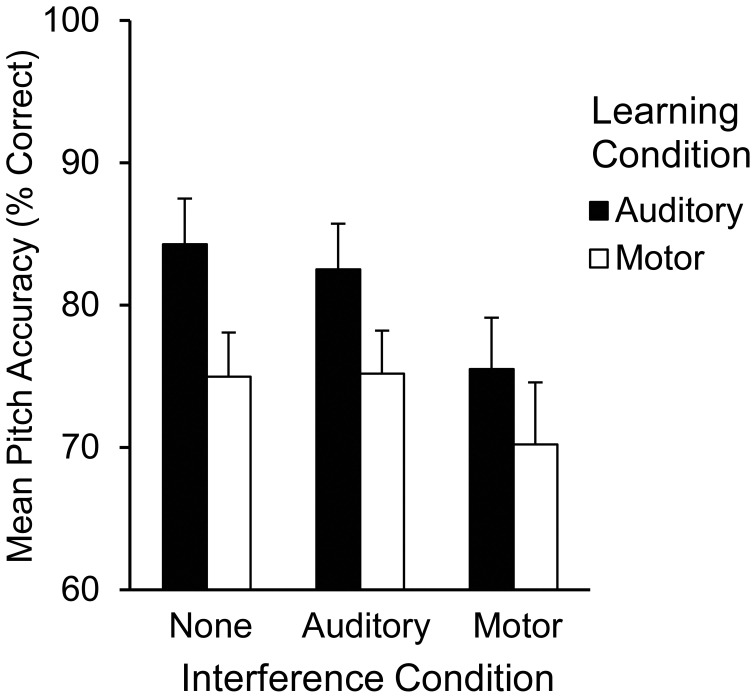
**Mean pitch accuracy (% correct pitches) during Recall by learning condition and interference condition in Experiment 2**.

The alpha level was set at 0.05 for all tests. Pitch accuracy during Recall was assessed for each trial by calculating the percentage of pitches performed correctly from memory out of 12 pitches. Mean pitch accuracy by learning and interference condition is shown in Figure 3. A two (learning condition: auditory, motor) by three (interference condition: none, auditory, or motor) repeated measures ANOVA on mean pitch accuracy, including auditory and motor imagery scores as covariates, revealed a main effect of learning condition [*F*_(1, 20)_ = 14.72, *p* < 0.01] such that pitch accuracy following auditory learning (*M* = 80.60%, *SEM* = 2.60) was higher than pitch accuracy following motor learning (*M* = 73.40%, *SEM* = 3.20); a main effect of interference condition [*F*_(2, 40)_ = 7.53, *p* < 0.01] revealed that pitch accuracy at Recall was lower in the presence of motor interference (*M* = 72.80%, *SEM* = 3.40) than in the presence of no interference (*M* = 79.50%, *SEM* = 2.60; *T*ukey's HSD = 6.66, *p* < 0.05). Participants' accuracy on the motor interference task was high (91.55% correct key-presses per trial), indicating that they performed the motor interference task correctly. There was no interaction between learning and interference conditions. A main effect of auditory imagery [*F*_(1, 20)_ = 6.69, *p* < 0.05] revealed that higher auditory imagery scores corresponded to higher pitch accuracy scores overall at Recall; there was no significant main effect of motor imagery or interaction between auditory and motor imagery. The parameter estimates for auditory and motor imagery in each condition (Table 2) reveal that higher auditory imagery scores significantly predicted higher pitch accuracy during Recall in all auditory learning conditions, across interference manipulations at Recall.

**Table 2:** **Experiment 2: Parameter estimates for auditory and motor imagery effects by condition**.

**Learning**	**Interference**	**Parameter**	**B**	***SE***	***t***	**Sig.**
Auditory	None	Intercept	0.84	0.03	31.13	<0.001
		**Auditory Imagery**	0.30	0.11	2.79	0.011^*^
		Motor Imagery	0.11	0.12	0.95	0.356
		Auditory^*^Motor Imagery	0.70	0.42	1.69	0.107
	Auditory	Intercept	0.82	0.03	29.33	<0.001
		**Auditory Imagery**	0.33	0.11	2.97	0.008^*^
		Motor Imagery	0.09	0.12	0.74	0.466
		Auditory^*^Motor Imagery	0.21	0.43	0.48	0.636
	Motor	Intercept	0.75	0.03	24.02	<0.001
		**Auditory Imagery**	0.35	0.13	2.78	0.012^*^
		Motor Imagery	0.13	0.14	0.94	0.357
		Auditory^*^Motor Imagery	0.56	0.48	1.16	0.261
Motor	None	Intercept	0.75	0.03	25.02	<0.001
		*Auditory Imagery*	0.24	0.12	2.003	0.059
		Motor Imagery	−0.09	0.13	−0.71	0.488
		Auditory^*^Motor Imagery	0.20	0.46	0.42	0.676
	Auditory	Intercept	0.75	0.03	24.76	<0.001
		Auditory Imagery	0.17	0.12	1.43	0.168
		Motor Imagery	0.06	0.13	0.48	0.638
		Auditory^*^Motor Imagery	0.22	0.47	0.48	0.639
	Motor	Intercept	0.70	0.043	16.20	<0.001
		*Auditory Imagery*	0.30	0.17	1.76	0.094
		Motor Imagery	−0.12	0.19	−0.64	0.530
		Auditory^*^Motor Imagery	−0.14	0.67	−0.20	0.840

***Bold***,

* **p<0.05**;

*Italics, p < 0.10*.

To further assess the relationship between participants' imagery abilities and other behavioral indices, pianists' auditory and motor imagery scores were correlated with each other, with years of piano instruction, self-rated sight-reading ability, age at which participants started playing piano, and number of hours they currently practice piano; none of the correlations reached significance (*p's* > 0.05). Last, we compared performance across experiments; neither pitch accuracy at Recall nor auditory or motor imagery scores in Experiment 2 differed from those of Experiment 1 (*p's* > 0.05). Thus, cross-experiment differences in how auditory and motor imagery influenced Recall cannot be attributed to cross-experiment differences in imagery scores.

### Temporal variability during recall

To examine how learning and interference conditions influenced performance timing during Recall trials, temporal variability (CV of quarter-note IOI) of performance during Recall trials was examined. Again, a subgroup of 12 subjects with the highest pitch accuracy (mean pitch accuracy per trial at Recall = 89.03%) was examined; only pitch-perfect Recall trials were included in the analysis. A two (learning condition) by three (interference condition) repeated measures ANOVA on CV measures, including auditory and motor imagery scores as covariates, revealed a main effect of interference [*F*_(2, 16)_ = 4.14, *p* < 0.05] such that temporal variability was smaller in the presence of motor interference (*M* = 0.037, *SEM* = 0.005) than in the presence of no interference (*M* = 0.043, *SEM* = 0.006) or auditory interference (*M* = 0.045, *SEM* = 0.004). The presence of an isochronous sequence produced by the left hand in the motor interference condition therefore improved the temporal regularity of the right hand. A significant interaction between interference and auditory imagery scores [*F*_(2, 16)_ = 6.33, *p* < 0.01] revealed that higher auditory imagery scores predicted greater temporal regularity in conditions with auditory interference; parameter estimates indicated significant negative unstandardized coefficients associated with CV measures in only the two auditory interference conditions (auditory learning*—*auditory interference: *B* = −0.064, *p* < 0.05; motor learning*—*auditory interference: *B* = −0.081, *p* < 0.05), indicating that as auditory imagery abilities increased, temporal variability of performance at Recall decreased in the presence of auditory interference.

## Discussion

Pianists' imagery abilities modulated the influence of type of learning (auditory or motor) but not type of interference (auditory, motor, or none) on pitch accuracy during performance from memory (Recall) in Experiment 2, when interference was introduced during Recall. Auditory imagery abilities did interact with interference conditions to influence temporal variability at Recall; pianists with high auditory imagery skill performed less variably in the presence of auditory interference. Motor imagery had no influence on overall pitch accuracy or temporal variability at Recall and did not interact with learning or interference conditions in Experiment 2. Higher auditory imagery skill was associated with higher pitch accuracy during Recall overall, and pianists with high auditory imagery showed higher pitch accuracy than pianists with low auditory imagery following auditory learning conditions, further suggesting that auditory imagery influenced pitch accuracy by modulating encoding. These findings suggest that auditory imagery abilities modulate memory retrieval as well as encoding. Similar to the findings of Experiment 1, pianists demonstrated higher pitch accuracy following auditory learning than motor learning. In contrast to Experiment 1, pianists performed with greater temporal regularity when motor interference was presented during Recall. Because the motor interference sequence was isochronous, its regular beat, produced by the left hand, may have aided the temporal regularity of the rhythmically varying stimuli produced by the right hand, similar to previous findings of reduced temporal variability in bimanual versus unimanual tapping (Helmuth and Ivry, 1996). These results further demonstrate that expert performers rely more on auditory learning than motor learning to perform accurately from memory, and the results additionally suggest that the motor interference task at Recall decreased pitch accuracy while aiding temporal regularity.

## General discussion

Musicians' imagery abilities modulated both memory encoding and retrieval of musical sequences. Individual differences in imagery abilities predicted how well pianists performed musical sequences from memory when different types of information were available during encoding (auditory or motor learning conditions), and when irrelevant information (auditory or motor) was presented during encoding (Experiment 1) or during retrieval (Experiment 2). Both motor and auditory imagery abilities influenced how accurately pianists performed pitch sequences from memory: higher imagery skills predicted higher pitch accuracy at Recall across learning and interference conditions of Experiment 1, when interference was introduced during encoding. Auditory imagery modulated encoding effects on pitch accuracy at Recall (Experiment 1): higher auditory imagery skills predicted higher pitch accuracy following auditory learning with interference and motor learning with motor or no interference. Auditory imagery abilities also influenced pianists' pitch accuracy of musical sequences when interference was introduced during Recall (Experiment 2) and modulated retrieval effects on temporal regularity (Experiment 2): higher auditory imagery abilities predicted greater temporal regularity in the presence of auditory interference at Recall. Motor imagery aided pitch accuracy at Recall when interference was present during encoding (Experiment 1) but not when interference was present during retrieval (Experiment 2). Overall, pianists performed pitches more accurately from memory following auditory learning than motor learning, and less accurately from memory when motor interference was present at Learning or at Recall. We discuss the implications of each of these findings in turn.

### Auditory and motor imagery

Auditory and motor imagery abilities influenced performers' encoding and retrieval of musical sequences in unique ways. The two imagery measures did not correlate with one another in this study or in previous studies (Highben and Palmer, 2004; Brown and Palmer, 2012). This finding is consistent with neural models of auditory-motor integration in speech which suggest that auditory and motor processes are partially distinct in skilled behavior (Hickok and Poeppel, 2004; Warren et al., 2005). The measures of auditory and motor imagery reported here may therefore reflect distinct cognitive abilities that engage different neural networks such as secondary auditory cortex for auditory imagery (Zatorre and Halpern, 2005) and premotor and parietal regions for motor imagery (Jeannerod, 2001). The distinct contributions of auditory and motor imagery in the current study therefore suggest that these abilities play different roles in sensorimotor learning; auditory imagery ability may support auditory encoding and motor imagery ability may support general performance of motor tasks from memory. Although auditory and motor imagery may be mediated by different systems, these systems may interact in tasks that require sensorimotor coupling (Lotze et al., 2003; Baumann et al., 2007) or executive processes such as working memory (Daselaar et al., 2010). Auditory and motor imagery abilities also did not correlate with measures of musical experience, as also reported previously (Highben and Palmer, 2004; Pecenka and Keller, 2009), suggesting that auditory and motor imagery abilities do not simply reflect greater musical experience.

Auditory imagery abilities influenced music encoding by modulating the influence of motor learning on subsequent pitch accuracy. High auditory imagery skill corresponded to higher pitch accuracy following motor learning with motor or no interference, suggesting that skilled auditory imagers may compensate for the missing auditory information that was not present during encoding. This finding is consistent with previous research demonstrating that skilled auditory imagers perform more accurately from memory (Highben and Palmer, 2004) and have better auditory recognition for sequences learned without auditory feedback (Brown and Palmer, 2012). This finding is also consistent with previous demonstrations of auditory imagery's activation of secondary auditory cortical regions (Zatorre and Halpern, 2005), which may be a neural mechanism by which high auditory imagers generate the subjective experience of missing auditory information during encoding. Auditory imagery abilities thus appear to aid accurate encoding of music by compensating for missing information.

Auditory imagery abilities also influenced music encoding by modulating the influence of auditory learning with interference on subsequent pitch accuracy. High auditory imagery skill predicted higher pitch accuracy following auditory learning in the presence of an additional motor task or auditory stimulus. Similar to previous dual-task designs (Li et al., 2001; Doumas et al., 2009), competing auditory or motor information may have increased working memory demands during encoding. Skilled auditory imagers may have overcome these increased demands through an ability to maintain or manipulate melodies in working memory while inhibiting irrelevant information; this enhanced working memory capacity may have helped skilled auditory imagers encode music more accurately. This explanation is consistent with previous studies suggesting that visual and auditory imagery vividness utilizes working memory (Baddeley and Andrade, 2000) as well as previous studies demonstrating that auditory, motor, and visual imagery engages prefrontal brain regions (Jeannerod, 2001; Daselaar et al., 2010; Herholz et al., 2012). The idea that skilled imagers are better able to maintain or manipulate information in working memory in the face of increased cognitive demands has been demonstrated for visual imagery, whereby individuals who are skilled in visuo-spatial imagery show greater spatial working memory (Sims and Hegarty, 1997) and demonstrate better mathematical problem-solving abilities than less-skilled spatial imagers (Hegarty and Kozhevnikov, 1999). Although previous findings demonstrated that high auditory imagery abilities hurt memory following competing auditory information at encoding (Brown and Palmer, 2012), that study presented interference sequences that were highly related to the test melodies (interference and test melodies differed only in the temporal onsets and offsets of pitch events). Pianists' sensitivity to interference may therefore depend on the degree to which interfering information is related to the task-relevant information. Auditory imagery abilities thus appear to aid accurate encoding of music by decreasing sensitivity to interfering information.

Auditory imagery abilities may engage a network of functionally linked auditory and pre-frontal brain regions that can be recruited to generate and maintain task-relevant auditory representations. The ability for skilled auditory imagers to engage this network may reflect both task-specific imagery advantages (Daselaar et al., 2010) and task-general, individual-specific advantages (Herholz et al., 2012). Although current findings do not distinguish these possibilities, they do suggest two complementary mechanisms by which imagery abilities aid performance: by completing missing information (Highben and Palmer, 2004; Brown and Palmer, 2012), and by decreasing sensitivity to interfering information.

Auditory imagery abilities also influenced performers' retrieval abilities. The effects of interference on the temporal regularity of performance during Recall were modulated by auditory imagery ability; high auditory imagery skill predicted greater temporal regularity in the presence of auditory interference during Recall. This benefit on temporal regularity at retrieval may arise from several sources. Competing auditory information presented at Recall may have increased working memory demands during retrieval. An enhanced capacity for skilled auditory imagers to maintain encoded melodies in working memory may have helped those skilled imagers inhibit irrelevant auditory information at retrieval and thus perform from memory with greater temporal regularity. As well, the metronomic regularity of the auditory interference melody may have aided the temporal regularity of execution; this interpretation is supported by the higher temporal regularity measured during the motor interference condition, and these findings are similar to previous demonstrations that better auditory imagery correlates with better sensorimotor synchronization abilities (Pecenka and Keller, 2009). Thus, the current findings further extend previous findings of imagery's influence on encoding (Highben and Palmer, 2004; Brown and Palmer, 2012) to influences on retrieval.

In sum, auditory imagery abilities modulated encoding effects on pitch accuracy (Experiment 1) and modulated retrieval effects on temporal regularity (Experiment 2). Together, these findings suggest that auditory imagery aided performers in learning correct pitch sequences at encoding and in executing sequences with greater temporal regularity at retrieval. Skilled auditory imagers may be more adept than less skilled imagers at forming and maintaining correct pitch-sequence representations at encoding and at suppressing irrelevant information both at encoding and at retrieval, which may aid their temporal execution at retrieval. This explanation is consistent with previous evidence suggesting that both pitch and temporal information in melodies is represented in auditory imagery (Zatorre and Halpern, 1993; Halpern and Zatorre, 1999; Janata and Paroo, 2006), and that imagining familiar auditory sequences engages motor behaviors such as covert articulation (Brodsky et al., 2003; Aleman and van't Wout, 2004) and motor networks such as the supplementary motor area (Halpern and Zatorre, 1999; Zatorre and Halpern, 2005; Leaver et al., 2009). Auditory imagery abilities may therefore influence not only auditory processes but also motor or auditory-motor mapping processes (Baumann et al., 2007). Auditory imagery abilities may engage auditory and motor processes differently at encoding, when correct pitch sequences must be maintained in working memory, versus retrieval, when temporal features of melodies must be executed. In addition, memory retrieval may involve continued encoding or even re-encoding to some extent, which may involve processes distinct from initial encoding and may place distinct demands on imagery abilities, an avenue for future research.

Although greater motor imagery abilities corresponded to higher pitch accuracy overall across learning and interference conditions at encoding (Experiment 1), motor imagery did not influence pitch accuracy in Experiment 2 or interact with encoding or retrieval effects on pitch accuracy or temporal regularity (Experiments 1 and 2). One explanation is that interference introduced during retrieval (Experiment 2) may have diminished any motor imagery effects that occurred at encoding or retrieval, thus yielding no motor imagery influence when interference was introduced at retrieval. Overall, the results suggest that motor imagery abilities may have general rather than specific influences on encoding and retrieval of music, and these influences may be enhanced by interference at encoding and diminished by interference at retrieval.

### Auditory/motor learning and interference

The current findings are the first to demonstrate that performance from memory is more accurate following auditory learning than motor learning. This is surprising, given that both auditory and motor information must be integrated in music performance, and the advantage of auditory learning was found regardless of whether interference was present or absent, across encoding and retrieval. These findings suggest that skilled performers rely more on learned auditory representations than on motor representations to perform auditory-motor sequences from memory. Skilled performers may rely more on feed-forward sensorimotor processes, in which representations of sensory outcomes directly activate motor commands, than on feedback sensorimotor processes, in which sensory feedback from online performance influences motor commands (Wolpert et al., 1995; Zatorre et al., 2007). In this sense, music performance may be similar to other motor skills such as speech that presumably rely on feed-forward motor control (Wolpert et al., 2001; Guenther, 2006). Auditory learning may also occur more quickly than motor learning; skilled performers may rely more on auditory representations at early stages of music encoding and motor representations at later stages. This idea is in line with previous findings demonstrating that motor learning aids later recognition of music, but only after sufficient amounts of practice (Brown and Palmer, 2012). If the pianists had experienced more practice during Learning trials in the current study, effects of motor learning might be predicted to be more pronounced. Additionally, as auditory and motor learning both involved musical notation (the presence of notation was kept constant across learning conditions), auditory-visual or visual-motor processes could have been engaged during learning conditions. Further research is needed to determine the influence of visual processes on auditory-motor encoding and retrieval processes.

The current findings also demonstrate that pitch accuracy at Recall was most impeded by motor interference regardless of whether interference was presented at encoding (Experiment 1) or retrieval (Experiment 2). The motor interference task required pianists to synchronize their movements in the left hand with their movements in the right hand that performed the test melody; although the motor interference sequence was in a lower register than the test melody, pianists had to integrate their left- and right-hand movements. In contrast, the auditory interference task required pianists to ignore the auditory interference melody; the auditory interference melody was presented in a higher register than the test melody and may have been easy to segregate perceptually from the test melody and thus have caused less interference (Bregman, 1990). Future studies may manipulate the relatedness of the interference and test melodies to further examine relative influences of motor and auditory interference.

In sum, we demonstrate that auditory and motor imagery abilities among performing musicians have distinct influences on encoding and retrieval of sensorimotor sequences. Auditory imagery abilities appeared to modulate encoding of pitch order in musical sequences and retrieval of temporal features of musical sequences. Motor imagery abilities yielded general, rather than specific, enhancements on performance of music from memory. The findings also demonstrate that auditory learning aids performance from memory more than motor learning, and that performance is most vulnerable to motor interference. This study further corroborates the importance of auditory representations in learning to perform auditory-motor sequences. Skilled performers thus vary widely in imagery capacities associated with their skill, and these capacities have distinct influences on learning and remembering sensorimotor sequences.

### Conflict of interest statement

The authors declare that the research was conducted in the absence of any commercial or financial relationships that could be construed as a potential conflict of interest.
